# Gastric adenocarcinoma metastasis to the rectum causing complete obstruction, a case report

**DOI:** 10.1093/jscr/rjad560

**Published:** 2023-10-21

**Authors:** Marine Bolliet, Mary Green, Amir Damadi

**Affiliations:** Department of Surgery, Ascension Providence Hospital-Michigan State University College of Human Medicine, Soutfield Campus, 16001 West Nine Mile Road, Southfield, MI 48075, United States; Michigan State University College of Osteopathic Medicine, East Lansing, MI, United States; Department of Surgery, Ascension Providence Hospital-Michigan State University College of Human Medicine, Soutfield Campus, 16001 West Nine Mile Road, Southfield, MI 48075, United States

**Keywords:** gastric adenocarcinoma, signet ring, rectal metastasis, case report

## Abstract

Gastric adenocarcinoma is a leading cause of mortality worldwide. The most common sites of metastases are the liver, peritoneum, lungs, and bones. Cases have been described in the colon and rectum, but are very rare. This case report describes a patient in remission from diffuse signet ring cell type gastric adenocarcinoma with resection and chemoradiation close to 10 years prior to presenting with a near-obstructing rectal mass that was consistent with metastatic gastric adenocarcinoma. Gastric cancer spreads via hematogenous, lymphatic, peritoneal seeding, or local recurrence pathways. Given the length of time between initial presentation and eventual metastasis, the theory of dormancy is discussed and proposed as a possible cause in the delay of metastasis to the rectum. This highlights the importance of maintaining a high index of suspicion for recurrence and metastasis in a patient with a history of gastric cancer, who presents with a new obstructing rectal mass.

## Introduction

Gastric cancer comprised 1.5% of newly diagnosed cancers in the United States in 2023 with 26 500 new cases and 11 130 deaths [1]. Recurrence after curative resection can complicate gastric cancer in up to 40.1% [2]. It is well documented that common sites of metastases include the liver, peritoneum, lung, but spread to the colon or rectum is rare [3]. Per the National Comprehensive Cancer Network, almost all recurrences occur by 5 years, and although controversial, recommended screening for recurrence typically does not exceed 5 years [4]. Although peritoneal spread is typically less common, in incidences of late recurrence, locoregional spread near the resection border and peritoneal spread to abdominal locations are more common than hematogenous spread [5]. Additionally, there is a higher incidence of peritoneal recurrence in patients with the more aggressive signet cell or undifferentiated gastric cancer types [6].

In this case report, we present a 59-year-old male with a history of diffuse signet ring cell type gastric adenocarcinoma in remission for close to 10 years after surgical resection, chemotherapy, and radiation who presents with a near complete obstructing mass in the distal rectum which, after pathologic analysis, was recognized as metastatic gastric adenocarcinoma.

## Case presentation

**Figure 1:**
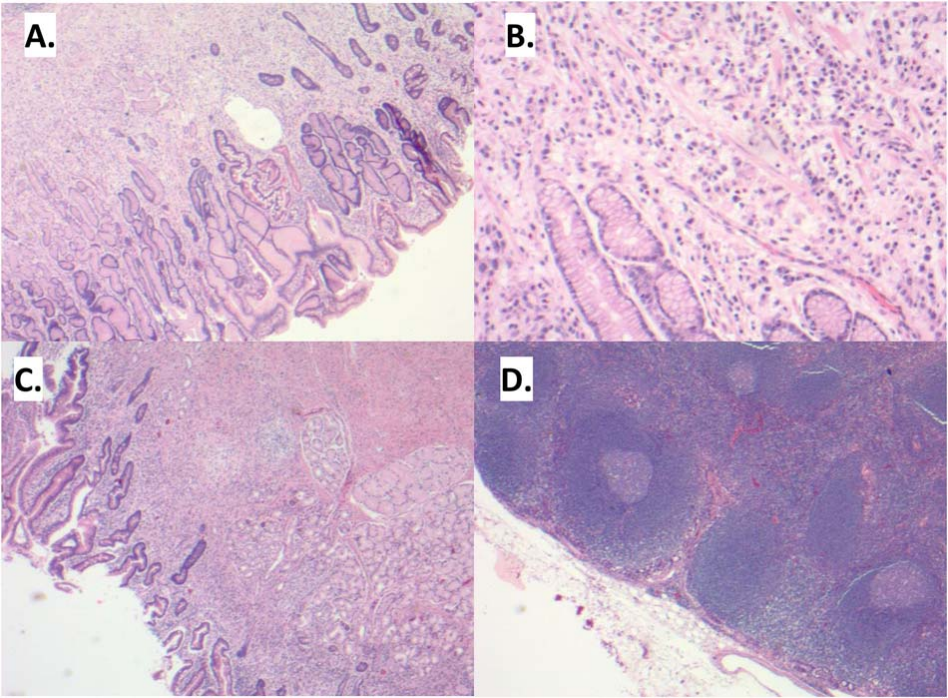
(A) Sections demonstrate an infiltrative lesion expanding the lamina propria of the gastric mucosa. Surface epithelium includes foci of intestinal metaplasia. (B) The infiltrative lesion is composed of signet ring tumor cells with irregular ovoid nuclei and abundant finely vacuolated cytoplasm present in a single cell distribution throughout all layers of the stomach wall including involvement of the serosa, greater and lesser omentum and duodenal mucosa (C). (D) Multiple lymph node metastases are also noted.

Patient is a 59-year-old male with a known history of diffuse signet ring cell type gastric adenocarcinoma (Fig. 1). He was managed with a near-total gastrectomy and roux-en-y gastrojejunostomy, chemotherapy, and radiation almost 10 years prior to his acute presentation. He has no family history of colorectal cancer. He was a two pack per day tobacco smoker, but quit when diagnosed with gastric cancer. Patient had been having loose stools since his near-total gastrectomy (112 months prior), but now presented with difficulty defecating, with little stool output. He complained of low midline abdominal pain, decreased appetite out of fear of inability to defecate, a 10 kg unintentional weight loss over 3 months, but no nausea or vomiting. Screening colonoscopy 2 years prior was unremarkable. Initial examination revealed an acute obstructing mass 4 cm from the anal verge. A computed tomography scan demonstrated rectal wall thickening and large stool burden. Diagnostic endoscopy was unable to be completed due to severity of the stricture. CEA level at this time was normal, 2.8.

He subsequently underwent a subtotal colectomy with end ileostomy and mucous fistula due to stricturing of the previous gastrectomy causing a closed loop obstruction and overt dilation of the colon with multiple serosal tears of the cecum. He additionally underwent a flexible sigmoidoscopy and transanal biopsy for continued work up of the rectal mass.

**Figure 2:**
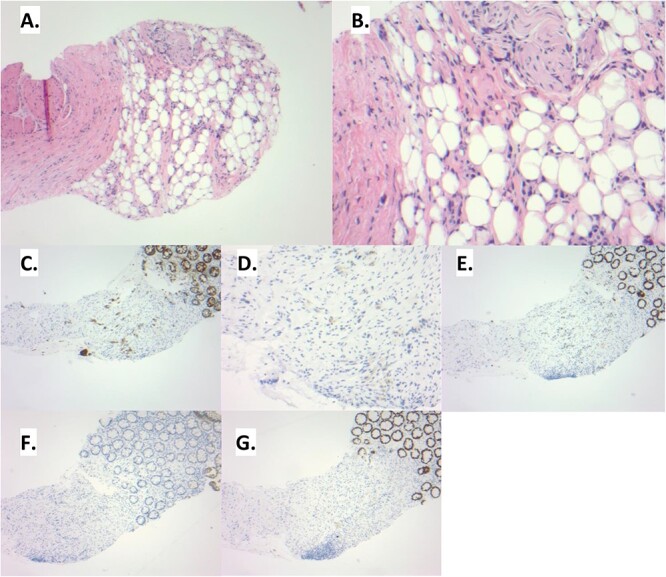
(A) The rectal biopsy shows distortion of the lamina propria. The overlying colonic epithelium appears benign. (B) Upon examination at higher power, signet ring cells can be noted within the loose connective and adipose tissue. These tumor cells are histologically similar to those seen in the prior gastrectomy. (C) This infiltrative pattern of tumor cells is highlighted by pankeratin. Additional immunohistochemistry is performed to delineate the site of origin of the signet ring cells. The tumor cells show an immunoprofile consistent with upper gastrointestinal tract origin: CK7 positive (D), CDX2 positive (E), CK20 negative (F), SATB2 negative (G).

Final pathology of the rectal mass demonstrated atypical epithelioid cells with signet ring cell features, compatible with gastric carcinoma (Fig. 2). Genetic testing was negative.

From this point, he required multiple admissions for failure to thrive and poor appetite. His enteral intake was supplemented with total parenteral nutrition to aid in his healing due to difficulty meeting nutritional requirements. Radiation oncology was consulted for palliative radiotherapy.

## Discussion

Gastric cancer rates are well reported and are increasing. Accompanying this, comes the subsequent increase in recurrence and metastasis. It has been shown that there is an up to 40% rate of recurrence in gastric cancer after curative resection [1]. Another study demonstrated that there is an even higher rate of recurrence in patients with a history of diffuse-type gastric adenocarcinoma, up to 65%, compared with intestinal-type, up to 41% [7]. It is evident that recurrence rates of gastric cancer after curative resection are much higher than would be expected, maintaining that close follow up following resection is crucial for recurrence monitoring.

Additionally, recurrence can present in a variety of ways and a high index of suspicion must be present. There are case reports that describe initial presentations of gastric adenocarcinoma as large bowel obstructions, suggesting that this malignancy is often diagnosed late and is insidious in nature [8]. Others report an aggressive and rapid spread through lymphatics, leading to diffuse metastases within a couple months [9]. Well described routes of metastases after curative resection of gastric adenocarcinoma is through lymphatics, peritoneal seeding, hematogenous spread, or local recurrence. Hematogenous spread causes shearing stress forces leading to only ~20% of the cells reaching an end organ [2, 10].

There are further theories that have been described, but have been incompletely studied. The theory of ‘dormancy’ suggests that malignant cells may spread at the time of initial malignant presentation, deposit in distal organs, remain dormant for a period of time, and after a physiologic stress they can become active again [11]. This would explain the often long latency period between initial malignancy and distal metastases. The patient described in this case report underwent resection, but was noted to have 7/15 perigastric lymph node metastases, as well as 9/10 positive greater omental lymph nodes. He underwent chemoradiation and was in remission until he presented 10 years later with an obstructing rectal lesion. This suggests that recurrence may happen over any time frame, and close monitoring is vital.

Due to his history of diffuse signet ring cell type gastric adenocarcinoma, in the presence of a new rectal mass, concern for gastric cancer recurrence was high. Given the near complete rectal obstruction, urgent stool diversion was performed, followed by further workup on the etiology of the mass. Diagnostic laparoscopy demonstrated no evidence of peritoneal seeding or metastases. After the pathology results were found to be consistent with gastric adenocarcinoma metastasis, the patient elected to proceed with chemotherapy and radiation.

In conclusion, gastric adenocarcinoma metastasis to the rectum is rare. In patients with a history of malignancy, a colonic or rectal obstruction secondary to a mass must be considered metastatic until proven otherwise. Gastric adenocarcinoma, especially diffuse signet ring cell type, is aggressive and can spread peritoneally, hematogenously, lymphatically, or through locoregional recurrence. It is critical to remember that prior curative resection does not negate the possibility of metastatic spread, even as far as 10 years later. The dormancy theory could potentially explain the prolonged period between initial presentation and metastasis. A high index of suspicion should be maintained to properly care for this rare, but important etiology of rectal stricture and obstruction.

## Data Availability

No datasets were generated or analyzed during the current study.
